# m^6^A RNA methylation regulators predict prognosis and indicate characteristics of tumour microenvironment infiltration in acute myeloid leukaemia

**DOI:** 10.1080/15592294.2022.2160134

**Published:** 2022-12-25

**Authors:** Xinai Liao, Ling Chen, Jingru Liu, Haoran Hu, Diyu Hou, Ruolan You, Xiaoting Wang, Huifang Huang

**Affiliations:** Central Laboratory, Fujian Medical University Union Hospital, Fuzhou, China

**Keywords:** AML, m^6^A, prognosis, tumour microenvironment, immunotherapy

## Abstract

Patients with acute myeloid leukaemia (AML) have poor prognoses and low overall survival (OS) rates owing to its heterogeneity and the complexity of its tumour microenvironment (TME). N6-methyladenosine (m^6^A) modification plays a key role in the initiation and progression of haematopoietic malignancies. However, the underlying function of m^6^A regulators in AML remains elusive. This study thoroughly analysed the m^6^A modification features of 177 AML patients based on 22 m^6^A regulators. Utilizing unsupervised clustering, we determined three distinct m^6^A modification patterns related to different biological functions, TME cell-infiltrating characteristics and clinical outcomes. Additionally, a risk score was constructed based on six m^6^A regulators-associated prognostic signatures and was validated as an independent and valuable prognostic factor for AML. Patients with a low-risk score exhibited better survival than those with a high-risk score. Many m^6^A regulators were aberrantly expressed in AML, among which *METTL14, YTHDC2, ZC3H13* and *RBM15* were observed to be associated with the OS of AML. In addition, these four m^6^A regulators were found to be noticeably related to the immune checkpoint inhibitor (ICI) treatments. Finally, we verified the expression levels of these four m^6^A regulators in AML and healthy samples and three groups of AML patients with different risk categories. Collectively, our study indicates that the m^6^A modification pattern is involved in TME immune-infiltrating characteristics and prognosis in AML. A better understanding of the m^6^A modification pattern will help enhance our knowledge of the molecular mechanisms of AML and develop potential prognosis prediction indicators and more effective immunotherapeutic strategies.

## Introduction

Acute myeloid leukaemia (AML) is a type of life-threatening haematological malignancy, the most common adult acute leukaemia with the poorest prognosis [1]. The survival rates for AML patients have not significantly improved over the past 30 years because the primary treatment strategies remained largely unchanged [2,3]. Although many studies have explored factors influencing AML tumorigenesis and progression [4–6], such knowledge has just begun to be translated into new therapies. Thus, the identification of potential markers will improve the treatment and prognosis of AML patients.

It is clear that epigenetic regulation, such as RNA methylation, contributes to tumorigenesis and progression in AML [7,8]. In eukaryotic mRNAs, N6-methyladenosine (m^6^A) is the most common modification, regulated by methyltransferases (‘writer’), demethylases (‘erasers’) and binding proteins (‘reader’). M^6^A RNA methylation is involved in many cellular processes such as cell cycle regulation, differentiation and tumorigenesis [9,10]. In numerous studies, m^6^A regulators have been found to play a critical role in a variety of biological functions in vivo, which engage in the development of many diseases including cancer [11,12], neurological diseases [13,14] and embryonic retardation [15]. In recent years, many researchers have focused on the effects of m^6^A regulators on the progression of AML. For example, *METTL3* inhibits myeloid differentiation by promoting the expression of *BLC2, c-MYC* and *PTEN* [16], and poor survival of AML patients is attributed to mutations in the gene regulated by the m^6^A modification [17]. The RNA demethylase *FTO* has been found to reduce aerobic glycolysis in leukaemia cells [18]. Likewise, YTHDF2 is overexpressed in human AML and prevents AML cells from apoptosis by inhibiting TNFR2 [19]. These findings indicate that some m^6^A regulators can potentially improve the therapeutic and predict prognosis of AML patients.

Several investigations have demonstrated that the tumour immune microenvironment (TIME) plays an essential role in the diagnosis and treatment of cancer [20,21]. The TIME may affect the patient’s response to immune checkpoint inhibitor (ICI) treatments, including those targeting programmed death-ligand 1 (*PD-L1*), programmed death 1 (*PD1*), as well as CTL–associated protein 4 (*CTLA4*) [22]. In addition, it is essential to note that m^6^A regulators may affect the progression and immunotherapy of tumours by adjusting TIME [23]. It is reported that m^6^A methylation modification patterns correlate with TME infiltration characteristics in gastric cancer [24]. Han et al. found that *YTHDF1* can prolong the immune response to neoantigens [25]. It suggests that *YTHDF1* may be a possible therapeutic target and a key player in tumour immune evasion. However, few studies have been conducted on m^6^A regulators for predicting prognosis and evaluating immunotherapy of AML. Therefore, studying the function of m^6^A regulators in AML may provide a new perspective for selecting more effective therapies and providing better prognoses for AML patients.

In our present study, we comprehensively analysed the 22 m^6^A regulators using the integrated data of AML patients and normal individuals from the Cancer Genome Atlas (TCGA), Gene Expression Omnibus (GEO) and University of California Santa Cruz (UCSC). Here, we identified three different patterns of m^6^A modification, which had obvious differences in molecular mechanisms, prognoses and TME cell infiltration characteristics. Furthermore, based on m^6^A regulators, we successfully constructed a scoring tool called risk score, which displayed reliability and sensitivity in predicting the prognosis of AML patients. Additionally, we screened out four differential expression and prognosis-related m^6^A regulators (*METTL14, YTHDC2, ZC3H13* and *RBM15*) through differential expression analysis and survival analysis, examined the therapeutic benefits of ICI therapy in AML patients with different *METTL14, YTHDC2, ZC3H13* and *RBM15* expression levels. Finally, we verified the expression of these four m^6^A regulators in clinical AML samples.

## Materials and methods

### Isolation of peripheral blood mononuclear cells (PBMCs)

The samples of humans were collected from 30 patients with newly diagnosed AML and 15 healthy donors at Fujian Medical University Union Hospital, Fuzhou, China. Details on AML patient samples are shown in Table 1 and Table S1. The study was approved by the Committee for the Ethical Review of Research, Fujian Medical University Union Hospital and informed consent was obtained from all the patients. PBMCs were isolated through the Ficoll gradient.

**Table 1.** Details on AML patient samples.

Details on AML patient samples					
NO.	Gender	Age	WBC(×10^9^/L)	FAB	Mutation Status
AML#1	Female	44	1	M0	None
AML#2	Male	62	15.45	M5	CBL; KIT
AML#3	Male	43	4.08	M2	None
AML#4	Male	39	2.8	M5	PTPN11
AML#5	Female	47	6.59	M5	CEBPA
AML#6	Male	45	25.79	M5	TP53; TET2; CEBPA
AML#7	Male	54	1	M5	WT1
AML#8	Male	68	0.76	M2	None
AML#9	Male	34	38.12	M5	NPM1; NRAS; FLT3-TKD
AML#10	Female	67	1.16	M5	DNMT3A; NPM1; PTPN11
AML#11	Male	59	3.18	M2	FLT3-ITD; NPM1; IDH2
AML#12	Male	34	67.5	M5	CBFb-MYH11
AML#13	Male	56	1.81	M5	None
AML#14	Female	54	1.97	M5	BCR/ABL1
AML#15	Male	14	92.5	M5	FLT3-TKD; RUNX1; PTPN11; PHF6

### AML dataset acquisition and processing

Raw RNA-seq-based data, clinical information of 151 AML samples, and somatic mutation data were downloaded from the TCGA database (https://portal.gdc.cancer.gov/) using the ‘TCGAbiolinks’ package [26], which was specifically developed for integrative analysis of GDC data. Subsequently, RNA-seq data (FPKM values) were then converted to transcripts per kilobase million (TPM) values using the ‘limma’ package [27]. Additionally, other two RNA-seq data [GSE9476 (38 normal and 26 AML samples), GPL96 (HG-U133A) Affymetrix Human Genome U133A Array and GSE23312 GPL10107 (SMD Print_1094 Homo sapiens)] were obtained from the GEO database (https://www.ncbi.nlm.nih.gov/geo/). Gene expression profile matrix files from all three databases were obtained from raw RNA-seq data using Perl [28]. The copy number data of AML was obtained from the UCSC dataset (https://xenabrowser.net/datapages/).

### Somatic mutation and copy number analysis of m^6^A regulators

To analyse the mutations and copy number variations (CNVs) of m^6^A regulators in AML samples, somatic mutation data was obtained from the TCGA database and further was analysed using the ‘Perl’ and the ‘maptools’ package [29]. The copy number of m^6^A regulators was evaluated using the R software (version 4.1.1) and the Perl. The differential expression of m^6^A regulators in normal and AML samples were analysed using the R software. The Pearson correlation algorithm was used to analyse the correlation of m^6^A regulators with the occurrence and development of AML by using R software.

### Unsupervised clustering of 22 m^6^A regulators

22 regulators were acquired from the TCGA and GSE9476 datasets to identify different m^6^A modification patterns, including 8 writers (*METTL3, METTL14, METTL16, WTAP, VIRMA, ZC3H13, RBM15* and *RBM15B*), 1 eraser (*FTO*) and 13 readers (*YTHDC1, YTHDC2, YTHDF1, YTHDF2, YTHDF3, HNRNPC, FMR1, LRPPRC, HNRNPA2B1, IGFBP1, IGFBP2, IGFBP3* and *RBMX*). The established unsupervised clustering was used to determine different m^6^A modification patterns according to the expression of the 22 m^6^A regulators and to classify patients for further analysis. The ‘ConsensusClusterPlus’ and ‘limma’ packages were used to perform the abovementioned analyses, which were repeated 1000 times to ensure the stability of the classification [30]. Subsequently, the differential expression of m^6^A regulators in different phenotypes was analysed using the ‘limma’ [27], ‘reshape2’ and ‘Nagpur’ packages [31].

### Gene set variation analysis (GSVA) of the m^6^A regulators

Gene set variation analysis (GSVA) is a nonparametric and unsupervised method that is usually used to estimate changes in pathways and biological processes [32]. We then used the ‘GSVA’ package to analyse the different enrichments in biological processes between m^6^A modification patterns. The gene set ‘C2. Cp.kegg. V6.2. Symbols’ was downloaded from MSigDB for the GSVA database for running the GSEA analysis. A *p*-value < 0.05 was considered statistically significant.

### Survival analysis of m^6^A regulators in patients with AML

To investigate the prognostic value of m^6^A regulators, survival analysis was performed using clinical data obtained from the TCGA. Kaplan–Meier (KM) survival curves of m^6^A regulators were plotted using the ‘survival’ and ‘survminer’ packages [33], and differences in survival rate were assessed using a log-rank test threshold of *p*-value < 0.05.

### Identification of differentially expressed genes (DEGs) among distinct m^6^A phenotypes

The ‘limma’ package was used to screen for m^6^A phenotype-related DEGs among the three m^6^A modification patterns identified from the abovementioned analyses [27], with a threshold of adjusted *p*-value < 0.001.

### Pathway and gene ontology enrichments

To identify the functions of DEGs in AML. Gene Ontology (GO) and Kyoto Encyclopaedia of Genes and Genomes (KEGG) pathway analyses were performed using the ‘cluster Profiler’ package of R [34]. GO analysis can be divided into three categories as follows: biological processes (BPs), cellular components (CCs) and molecular functions (MFs). *p* < 0.05 was set as the cut-off for statistical significance.

### Construction of m^6^A regulators signature

To evaluate the relationship between m^6^A regulators and survival, univariate Cox regression analysis was performed to determine which m^6^A regulators were associated with survival (*p* < 0.05). We then used the least absolute shrinkage and selection operator (LASSO) Cox regression and multivariate Cox regression analysis to screen out six m^6^A regulators, which had the highest prognostic value for the formation of the prognostic signature. The risk score calculation is as follows:

$$m6A\left(riskscore\right)=\Sigma Incoef\left(m6A\;regulators\right)\;\;\;\;\;\;\;\;\;\;\;\;\;\;\;\;\;\;\;\;\;\;\;\;\;\;\;\;*expr\left(m6A\;regulators\right)$$

AML patients were classified into high- and low-risk groups based on the median risk score. We utilized the Kaplan-Meier method to analyse the survival of patients within the two groups. Then, univariate and multivariate Cox regression analyses were used to assess the independent prognostic value of the risk score. In addition, receiver operating characteristics (ROC) curve analysis was used to evaluate the accuracy and sensitivity of the risk prediction model.

### Evaluation of TME cell infiltration

Single-sample gene set enrichment analysis (ssGSEA) was used to quantify the relative abundance of infiltrating immune cells in the TME of AML using the ‘ssGSEA’ package. The gene set used to label each infiltrating immune cell type of TME was obtained from a study by Charoentong [35,36]. The gene set contains data regarding various human immune cell subtypes, including activated B cells, activated dendritic cells, T follicular helper cells, eosinophils and regulatory T cells. The enrichment fraction calculated was used to represent the relative abundance of infiltrating immune cells in each sample using ssGSEA. Then, the Wilcox test was performed to compare the expression of different immune cells in three m^6^A modification patterns. The Pearson correlation algorithm was used to analyse the correlation between immune cells and risk score in AML.

### Quantitative reverse transcription polymerase chain reaction (qRT-PCR)

The TRIzol reagent (Invitrogen) was used to extract total RNA from 11 pairs of samples and 15 AML samples (including favourable, intermediate and adverse). cDNA was synthesized using 5×All-in-One RT MasterMix with AccuRT (abm, Canada), and Eva Green 2×qPCR MasterMix-Low ROX (abm, Canada) was used to evaluate mRNA levels. The relative expression of m^6^A regulators were normalized to GAPDH and calculated by 2^−ΔCt^ method. The primer sequences are listed in Supplementary Table S2.

### Western blot analysis

Total proteins from 3 pairs of samples were extracted by RIPA buffer (Beyotime) containing protease and phosphatase inhibitor cocktail (Thermo). Protein concentration was determined using a BCA Protein Assay Kit (Thermo). Proteins were separated using SDS‑PAGE (10% gels) and transferred onto polyvinylidene difluoride (PVDF) membranes. Subsequently, the membranes were blocked with 5% BSA at room temperature for one hour and incubated with primary antibodies overnight at 4°C. The following day, the membranes were incubated with HRP-conjugated secondary antibodies (Beyotime, #A0208, #A0216) at room temperature for one hour. Chemiluminescence signals were visualized with a BeyoECL Star Kit (Beyotime).

### Flow cytometry

All FCM studies were performed using single-cell suspensions, and cells were stained in accordance with the standard protocols. Antibodies recognizing CD45-eFluor506 (#69045942, Thermo), CD4-Percp/Cyanine5.5 (#30050, Biolegend), CD8-Bv786 (#563823, BD Pharmingen,), CD3-FITC, CD16/CD56-PE (#A07735, Beckman) and CD19-PerCP5 (#302227, Biolegend) were used. Samples were analysed using a flow cytometer (BD Biosciences), and subsequent analysis was performed using FlowJo 10.1 software.

### Statistical analysis

The GraphPad Prism (version 9.2, GraphPad Software, La Jolla, CA, USA) was used for statistical analysis of all experimental data. The student’s t-test was performed for between-group comparisons. A *p*-value < 0.05 was considered statistically significant.

## Results

### The landscape of expression and genetic variation of m^6^A regulators in AML patients

**Figure 1.**
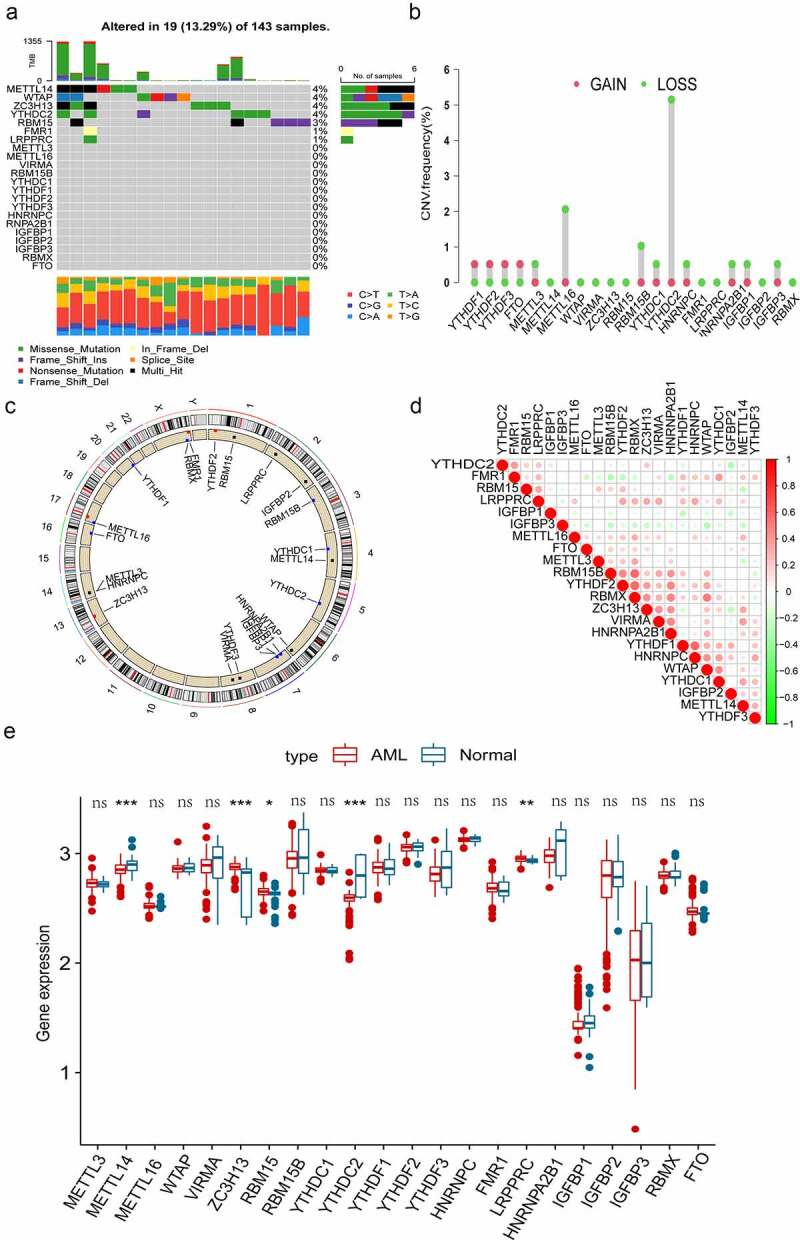
Genetic variation landscape and expression of m^6^A regulators in AML patients. (a) The frequency of mutations in 22 m^6^A regulators in 143 AML samples obtained from TCGA database. Each column represents individual patients. The upper bar plot represents TMB, and the number on the right represents the frequency of mutations in each regulator. The proportion of each type of variation is depicted in the right bar plot. The stacked bar plot below demonstrates the percentage of conversions in each sample. (b) The frequency of CNVs in m^6^A regulators in TCGA cohort. The height of the column represents the frequency of change. The green dots represent the deletion frequency, whereas the red dots represent the amplification frequency. (c) The location of CNVs in m^6^A regulators on 22 chromosomes in TCGA cohort. (d) Co-expression of m^6^A regulators analysed and visualized via Pearson correlation analysis. (e) Comparison of the expression of 22 m^6^A regulators in normal and AML samples. The colour of tumour samples is red, whereas that of normal samples is blue. The interquartile range of data is indicated by the upper and lower ends of the boxes. The median value is represented by the lines in the boxes. The statistical *p*-value is denoted by asterisks (*p < 0.05; **p < 0.01; ***p < 0.001).

In this study, there were 22 m^6^A regulators, including 8 writers, 1 eraser and 13 readers were identified. First, we evaluated the somatic mutations of these m^6^A regulators and found that only 19 (13.29%) of the 143 samples experienced m^6^A regulator mutations in AML. Among the 22 m^6^A regulators, *METLL14, WTAP, ZC3H13* and *YTHDC2* exhibited the highest mutation frequency, followed by *RBM15, FMR1* and *LRPPRC* (Figure 1a). We then performed the somatic CNV analysis, and the result revealed that most m^6^A regulators had copy number deletions except for *YTHDF1, YTHDF2, YTHDF3* and *FTO* (Figure 1b). Figure 1c shows the position of CNV changes of m^6^A regulators on the chromosomes. Correlation analysis discovered that most m^6^A regulators displayed close coordination in AML (Figure 1d). To further explore the value of m^6^A regulators in the pathogenesis and progression of AML. We examined the mRNA expression levels of the m^6^A regulators between normal and AML samples and observed that AML patients showed increases in the expression of *ZC3H13, RBM15* and *LRPPRC*, whereas there was a decrease in *METTL14* and *YTHDC2* than normal samples (Figure 1e). The above results indicated that the mutation and CNVs alteration of m^6^A regulators could influence their expression in AML. We also noted that an imbalance in the expression of m^6^A regulators might play a crucial role in the occurrence and progression of AML.

### Patterns of m^6^A methylation modification and its biological characteristics in AML

**Figure 2.**
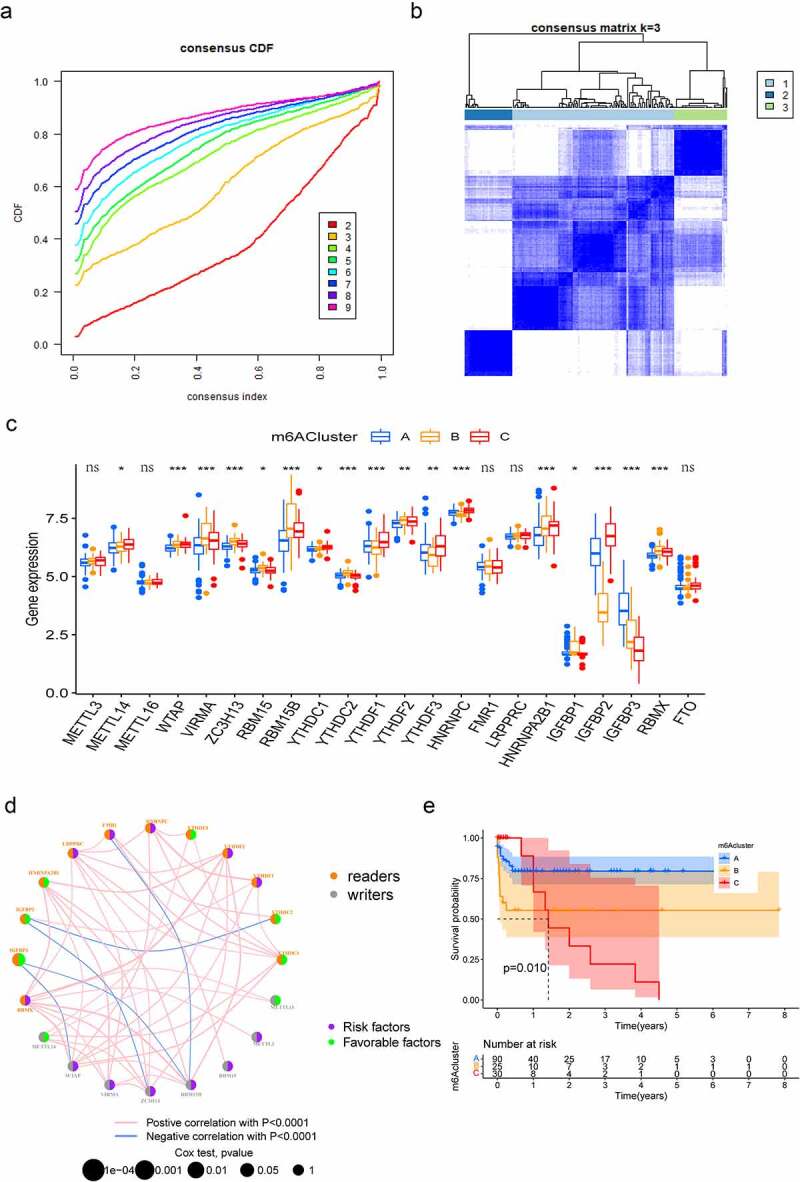
Patterns of m^6^A methylation modification. (a) Consensus CDF of TCGA cohort for k = 2–9. (b) Consensus matrices of TCGA cohort for k = 3. (c) Expression of 22 m^6^A regulators under three m6Aclusters. The lower and upper ends of boxes represent the interquartile range of values (*p < 0.05; **p < 0.01; ***p < 0.001). (d) Interactions among m^6^A regulators in AML. The size of the circles represents the effect of each regulator on prognosis. Purple dots in the circle represent prognostic risk factors; green dots in the circle represent prognostic protective factors. The lines connecting regulators represent their interactions, and the thickness represents the strength of correlation between regulators. Negative correlation is highlighted in blue, whereas positive correlation is highlighted in red. (e) Survival analyses for the three m^6^A modification patterns: Kaplan–Meier curves with log-rank *p*-values demonstrating significant survival differences among the three m^6^A modification patterns in 145 patients with AML (TCGA cohort), with 90 cases in m6Acluster-A, 25 cases in m6Acluster-B and 30 cases in m6Acluster-C. Overall survival was considerably higher in m6Acluster-A than in the other two m6Aclusters.

To comprehensively analyse the role of multiple m^6^A regulators in AML, the ‘ConsensusClusterPlus’ R package was used to identify distinct m^6^A modification patterns based on the expression of 22 m^6^A regulators and divide patients for further exploration. As shown in Figure S1A, B and Figure 2a, k = 3 was found to be optimal clustering stability from k = 2 to 9 based on the similarity displayed by the expression levels of m^6^A regulators. A total of 177 patients with AML were clustered into three subtypes, namely, cluster-A, cluster-B and cluster-C, based on the expression levels of the m^6^A regulators (Figure 2b). The principal component analysis (PCA) results revealed significant differences in the transcriptome profiles of different modification patterns (Figure S1C). Moreover, we analysed the relationship between the clinical characteristics and gene expression of three subgroups and found that the discrepancy seemed not obviously (Figure S1D). To further investigate the potential biological behaviours of the different m^6^A modification patterns, we performed GSVA enrichment analysis. M6Acluster-A was significantly enriched in carcinogenic activation pathways including ECM receptor interaction, TGF beta signalling pathway and cell adhesion (Figure S2A, B). M6Acluster-B displayed enrichment of pathways related to cytokine–cytokine receptor interaction, apoptosis, natural killer cell-mediated cytotoxicity, as well as T cell receptor signalling (Figure S2A, C) and m6Acluster-C was strongly linked to immune suppression (Figure S2B, C). Subsequently, the apparent differences in m^6^A regulators’ expression were observed in these three subgroups (Figure 2c). The m^6^A regulators network depicted the entire landscape of interactions among the m^6^A regulators and their relationship to AML prognosis. We found that not only the m^6^A regulators in the same functional category presented a remarkable correlation in expression, but also a significant correlation was shown among regulators in the reader and writer functional categories. (Figure 2d). To further clarify the intrinsic biological differences that led to distinct clinical phenotypes, we investigated the TME cell infiltration in three modification patterns. It was found that m6Acluster-A presented particularly rich in activated B cells, activated CD4^+^ T cells, CD56 bright natural killer cells and immature dendritic cells. M6Acluster-B showed the highest infiltration of immature B cells, natural killer cells, T follicular helper cells, type 1 T helper cells, type 17 T helper cells and type 2 T helper cells, whereas m6Acluster-C was characterized by immune suppression (Figure S2D). Our survival analysis revealed that the patients with the m6Acluster-A modification pattern had the best survival, and the survival of the patients with the m6Acluster-C modification pattern was the worst. However, the patients with m6Acluster-B modification pattern did not possess matching survival advantages (Figure 2e). Previous studies have proved that tumours with an immune rejection phenotype presented large numbers of immune cells that remain in the stroma surrounding tumour cell nests rather than penetrating their parenchyma [24]. These results suggested that the composition of m^6^A regulators may influence the establishment of distinct m^6^A modification patterns, tumour microenvironment, tumorigenesis and prognosis of AML.

### Generation of m^6^A phenotype-related DEGs and functional annotation in AML

**Figure 3.**
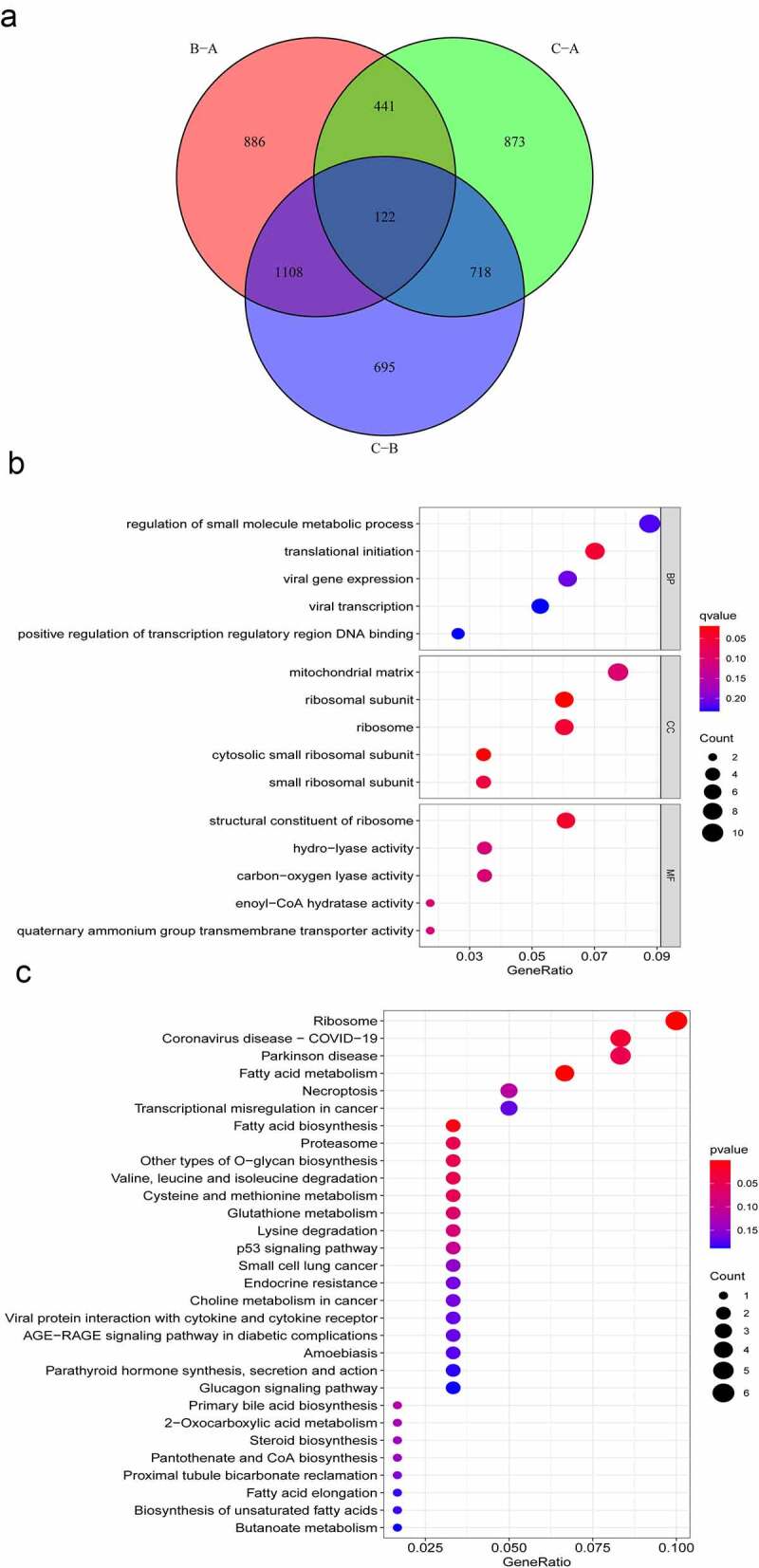
Functional annotation of m^6^A modification patterns in AML. (a) Venn diagram showing 122 m^6^A-related DEGs identified using the ‘limma’ package. (b) Functional annotation of DEGs using GO enrichment analysis. The colour depth of circles represents the q-value, and the size represents the number of genes enriched. (c) KEGG pathway analysis of DEGs. The colour depth of the circle represents the *p*-value, and the size represents the number of genes enriched.

To further explore the underlying biological procedures among the three m^6^A modification patterns, 122 m^6^A phenotype-related DEGs were identified through the ‘limma’ package (Figure 3a). The ‘clusterProfiler’ package was used to perform GO and KEGG analysis for these 122 m^6^A phenotype-related DEGs. BPs terms indicated that the DEGs were primarily enriched in ‘translational initiation.’ CCs ascribed to these DEGs mainly included ‘ribosomal subunit’ and ‘cytosolic small ribosomal subunit’ and the primary MFs included ‘structural constituent of ribosome’ (Figure 3b). The KEGG pathway enrichment analysis revealed that the DEGs were mainly enriched in pathways related to ‘fatty acid metabolism,’ ‘ribosome’ and ‘fatty acid biosynthesis’ (Figure 3c).

### The relationship between m^6^A regulators with overall survival in AML

**Figure 4.**
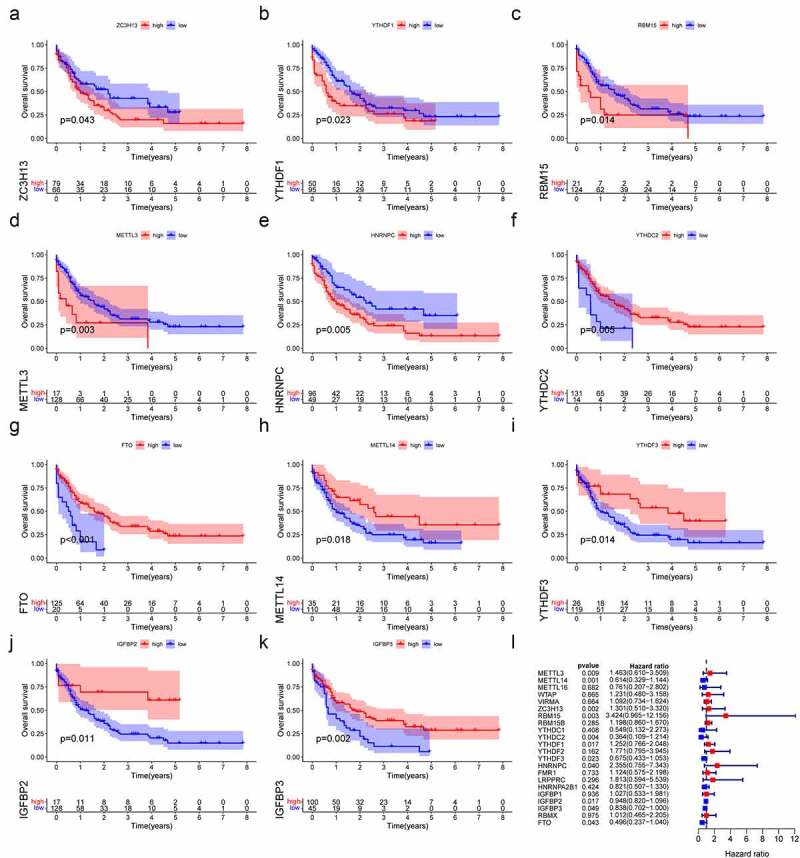
The relationship between m^6^A regulators with overall survival in AML. (a–k) Survival curve analysis of m^6^A regulators, which were significantly related to the prognosis of AML in TCGA database. (l) The forest plot shows the results of univariate Cox regression analysis.

Then, Kaplan-Meier survival curve analysis was used to detect the prognostic value of m^6^A regulators in AML patients. Based on the TCGA database, a total of 11 m^6^A regulators were verified to be significantly associated with AML prognosis. As shown in Figure 4a-k, *ZCH13H* (P = 0.043), *YTHDF1* (P = 0.023), *RBM15* (P = 0.014), *METLL3* (P = 0.003) and *HNRNPC* (P = 0.005) were remarkably related to worse prognoses in AML patients. On the contrary, *YTHDC2* (P = 0.043), *FTO* (P < 0.001), *METTL14* (P = 0.018), *YTHDF3* (P = 0.05), *IGFBP3* (P = 0.02) and *IGFBP2* (P = 0.011) were significantly associated with better outcomes. We also validated the prognostic value of *YTHDC2, METTL14, RBM15* and *ZC3H13* in AML based on the GSE23312 database (Figure S3). In summary, m^6^A regulators could have crucial roles in predicting clinical outcomes in AML patients.

### Construction and validation of prognostic signatures based on m^6^A regulators in AML

**Figure 5.**
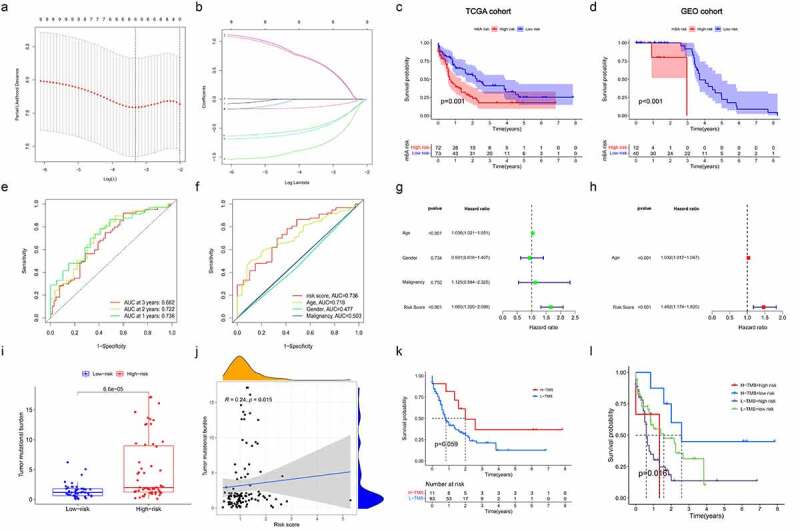
Construction and validation of prognostic signatures based on m^6^A regulators in AML (a, b) The process of developing the signature based on 11 m^6^A regulators and selected out six meaningful m^6^A regulators in AML. (c, d) The associated between risk score and OS of AML patients in TCGA and GEO databases. (e). Time-dependent ROC curves of the risk score and their AUC values at 1-, 2-, and 3-years. (f). The sensitivity ROC curves of the risk score and other clinical factors. (g, h) Multivariate and univariate Cox regression analyses of the clinicopathological features and risk score. (i, j) The correlation between TMB and risk score. (k) Kaplan–Meier survival curves of the low- and high-TMB groups in TCGA cohort. (l) Kaplan–Meier survival curves for subgroup patients stratified by both risk score and TMB.

To evaluate the value of 22 m^6^A regulators in predicting prognosis in AML precisely. First, we conducted univariate Cox regression analysis and found that *METTL3* (*p* = 0.009), *METTL14* (*p* = 0.001), *ZC3H13* ((*p* = 0.002), *RBM15* (*p* = 0.003), *YTHDC2* (*p* = 0.004), *YTHDF1* (*p* = 0.017), *YTHDF3* (*p* = 0.023), *YTHDF1* (*p* = 0.017), *HNRNPC* (*p* = 0.04), *IGFBP2* (*p* = 0.017), *IGFBP3* (*p* = 0.023) and *FTO* (*p* = 0.043) were significantly associated with OS (Figure 4l). Subsequently, only six m^6^A regulators (*RBM15, YTHDC2, YTHDF3, HNRNPC, IGFBP3* and *FTO*) were identified to be the independent prognostic genes from the 11 prognostic m^6^A regulators to develop the prognostic signature by the LASSO regression analysis and multivariate Cox regression analysis (Figure 5a,b; Table S3). And risk scores of AML patients were calculated using the coefficients obtained from LASSO regression analysis and multivariate Cox regression analysis. The equation was as follows: risk score = (0.9505 * *RBM15* expression) – (0.9642 * *YTHDC2* expression) – (0.6941 * *YTHDF3* expression) + (0.9479 * *HNRNPC* expression) – (0.1764 * *IGFBP3* expression) – (0.6830 * *FTO* expression). According to the median risk score, AML patients were classified into high-risk and low-risk groups. Then, Kaplan-Meier curve analysis was used to explore the prognostic value of risk groping in AML patients. The results showed that the low-risk subgroup had better survival outcome than the high-risk subgroup in the TCGA cohort (Figure 5c). Importantly, we used the GSE23312 AML datasets to verify the role of the prognostic signature in AML. As shown in Figure 5d, the risk-high subgroup was significantly relevant to worse prognosis, consistent with the result of the TCGA dataset. Moreover, ROC curve analysis was applied to estimate the specificity and sensitivity of the prognostic signature based on m^6^A regulators. Time-dependent ROC curves revealed that the areas under the curve (AUCs) at 1- and 2-years were 0.736 and 0.722 in the TCGA dataset, indicating good prediction performance of this prognostic model (Figure 5e). Univariate and multivariate Cox regression analyses were utilized to determine whether the risk score and clinical factors, such as age, sex and malignancy, may serve as independent prognostic indicators for AML patients (Figure 5g, h). The finding highlighted that age (*p* < 0.001) and risk score (*p* < 0.001) were independent prognostic factors for survival prediction. Moreover, the risk score’s sensitivity was higher than other clinical factors (figure 5f). Besides, we determined the relationship between risk score and tumour mutation burden (TMB) and how this related to patient outcomes. The risk score showed a positive correlation with TMB (Figure 5i, j). We further confirmed the prognostic value of TMB in AML patients and observed that the OS of AML did not significantly differ between the high- and low-TMB groups (Figure 5k). Stratified survival analyses suggested that the risk score might be a potential predictor independent of TMB for AML patients (Figure 5l). Taken together, the risk prediction model displayed satisfactory performance in predicting the survival of AML patients. However, whether these gene expression patterns could be used as tumour markers needs to be further verified in a large cohort of healthy subjects.

### The risk score is correlated with immune infiltration levels in AML

**Figure 6.**
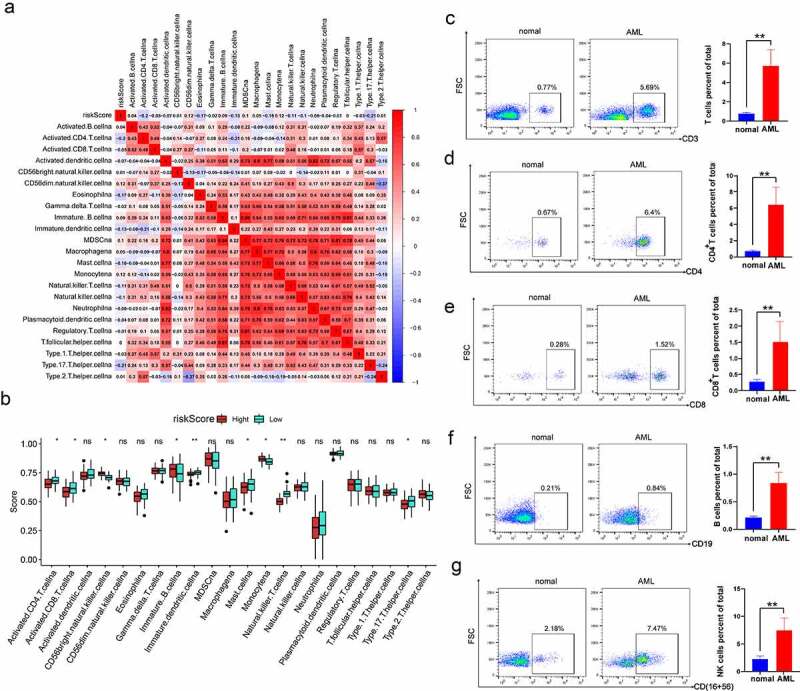
The risk score is correlated with immune infiltration levels in AML (a) Correlation between risk score and infiltrating cells in TME. Positive correlation is shown in red, and negative correlation is shown in blue. (b) Comparing the distributions of immune cells in both high- and low-risk groups in AML patients. (c-g) Flow cytometry analysis for the evaluation of the number of T cells, B cells and NK cells in healthy samples and AML samples. The asterisks denote the statistical *p*-value (*p < 0.05; **p < 0.01; ***p < 0.001).

Furthermore, the correlation of risk score with immune cell infiltration was evaluated in AML (Figure 6a). The finding revealed that risk score was negatively correlated with activated CD4 T cells, eosinophils, mast cells and type 17 helper cells. In addition, there was a close relationship among various immune cells. Specifically, MDSC was significantly positively associated with other immune cells. Besides, the Wilcoxon test was utilized to compare the distributions of immune cells in both high- and low-risk groups. We detected more immature B cells, Monocyte cells and CD56 bright natural killer cells in the high-risk score group. The low-risk score group had more activated CD8^+^ T cells, activated CD4^+^T cells and natural killer T cells (Figure 6b). To explore the characteristics of immune cell infiltration in clinical samples, we collected PBMCs from 5 AML patients and 5 healthy donors to detect the proportion of CD4^+^T cells, CD8^+^T cells, B cells and CD56^+^ NK cells. Compared with healthy samples, AML patients had an increased number of these immune cells (Figure 6c-g). These series of studies further demonstrated that m^6^A modification may play a vital role in regulating TIME and the m^6^A risk score may be used to evaluate the TIME features in AML.

### Correlation of m^6^A regulators and immunotherapy characteristics in AML

**Figure 7.**
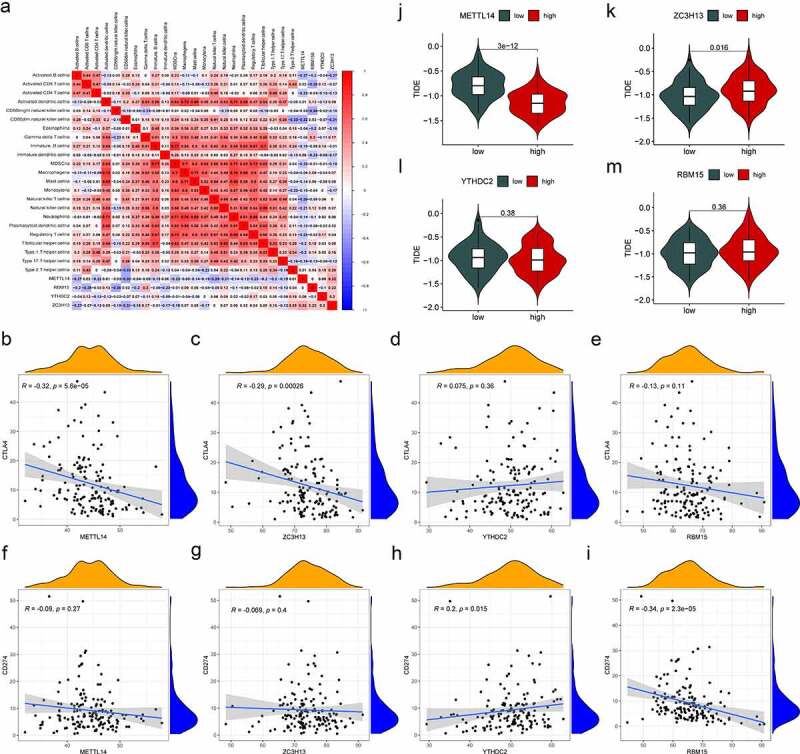
Correlation of m^6^A regulators and immunotherapy characteristics in AML. (a) The correlations between immune cell infiltration and the expression levels of candidate regulators. Positive correlation is represented in red whereas negative correlation is represented in blue. (b-i) Relationships between the expression of candidate regulators and *CTLA4* (b-e), and *PD-L1* (f-i). (j-m) Evaluation of the differences in TIDE predicting score between the high- and low-expression groups of candidate regulators. *p*-value (*p < 0.05; **p < 0.01; ***p < 0.001).

In the present study, four m^6^A regulators (*METTL14, YTHDC2, ZC3H13* and *RBM15*) were selected, which were differentially expressed and correlated with survival in AML. Then, we investigated the relevance of the characteristics of immunotherapy with these four regulators. The connection between the composition of immune cells and the expression of m^6^A regulators was analysed (Figure 7a). It was found that most immune cells were negatively correlated with *METTL14* expression and positively correlated with *ZC3H13*. Gene correlation analysis showed that the immune genes (*CTAL4* and *PD-L1*) and these four regulators had different correlations (Figure 7b-i). Among them, *CTAL4* was negatively correlated with *METTL14* and *ZC3H13*, while *CTAL4* was not significantly associated with *YTHDC2* and *RBM15. PD-L1* was positively related to *YTHDC2*, whereas *PD-L1* was negatively correlated with RBM15. Finally, tumour immune dysfunction and exclusion (TIDE) was used to evaluate the potential clinical benefits of immunotherapy in different expression levels of these four regulators (Figure 7j-m). A lower TIDE prediction score presented a lower possibility for immune evasion, suggesting that the patients could benefit more from ICI therapy [37]. We could find that the METTL14-low group had a higher TIDE prediction score than the METTL14-high group, implying that METTL14-high patients were more likely to benefit from ICI therapy than METTL14-low patients. In contrast, ZC3H13-high patients were less likely to benefit from ICI therapy than ZC3H13-low patients. These results indicated that *METTL14, YTHDC2, ZC3H13* and *RBM15* may affect immune responses and be a tool to assess immunotherapy response in AML.

### Verification of m^6^A regulators expression through qRT-PCR and western blot analysis

**Figure 8.**
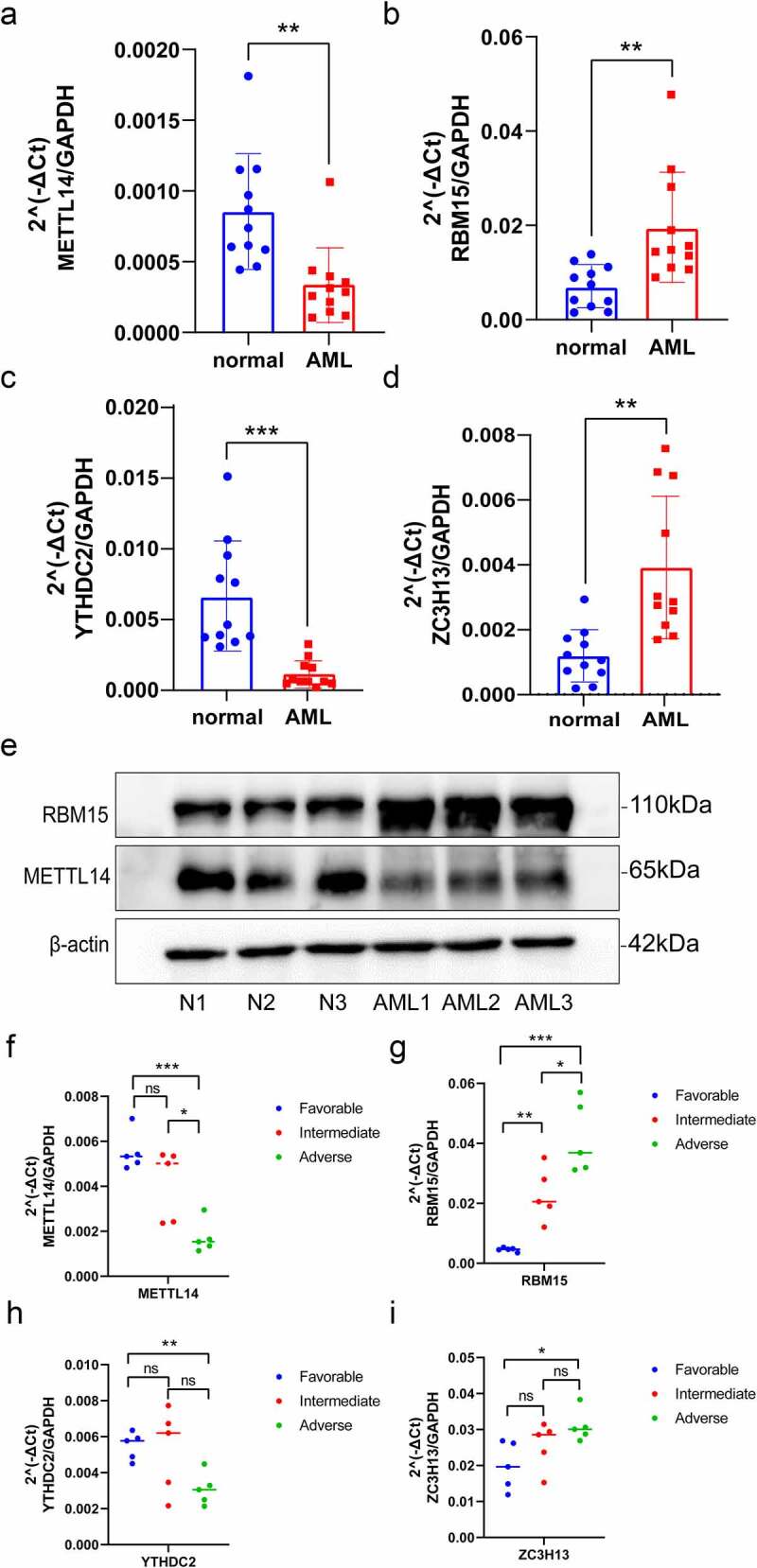
Validation of the expression of m^6^A regulators via qRT-PCR and Western blot analysis. (a–d) The mRNA expression of *METTL14, RBM15, YTHDC2* and *ZC3H13* between healthy and AML samples were determined via qRT-PCR. (e) METTL14 and RBM15 protein expression levels in three pairs of AML samples and healthy samples. (f-i) QRT-PCR examined the mRNA expression levels of *METTL14, RBM15, YTHDC2* and *ZC3H13* in three groups of AML patients with different risk categories. *p*-value (*p < 0.05; **p < 0.01; ***p < 0.001).

To verify the differential expression of *YTHDC2, METTL14, RBM15* and *ZC3H13* between AML and healthy samples, the total RNA was isolated from PBMCs for qRT-PCR (Figure 8a-d). Compared to healthy samples, *YTHDC2* and *METTL14* expression were significantly downregulated in AML samples, while *RBM15* and *ZC3H13* were upregulated in AML samples. Additionally, we found that the protein expression levels of RBM15 were significantly increased, and METTL14 were significantly decreased in AML samples by Western blot analysis (Figure 8e), which was consistent with the results of qRT-PCR. Then, we further investigate the relevance between these four genes’ expression levels and different AML patient groups. According to ‘2022 ELN risk classification by genetics at initial diagnosis’ [38], the AML patients were divided into three groups, including favourable, intermediate and adverse (Table S1). The total RNA was isolated from PBMCs for qRT-PCR. We discovered that the expression levels of *RBM15* and *ZC3H13* were significantly higher in the adverse risk compared with the favourable and intermediate risk of AML samples (Figure 8g, 8i). And the expression levels of *YTHDC2* and *METTL14* were significantly decreased in adverse risk of AML samples (figure 8f, 8h). Thus, these genes were verified successfully and showed good correspondence with the results of transcriptomic analysis, which yielded precise and reliable microarray results.

## Discussion

AML is a highly heterogeneous and fatal tumour associated with poor outcomes as a result of the high rate of recurrence [39]. In eukaryotes, m^6^A is the most common form of mRNA modification [40]. Increasing evidence demonstrates that m^6^A modification and its regulatory proteins play an essential role in various cancers, including leukaemia [41,42], brain tumour [43], breast cancer [44] and lung cancer [45]. Although there have been few reports in recent years on the role of m^6^A modification patterns in AML [46,47]. There is no report on biomarkers based on m^6^A regulators for AML prognosis prediction and immunotherapy evaluation. Additionally, the effect of m^6^A modification on the AML TIME has not been comprehensively understood. Therefore, integrating and reanalysing genomic profiles of m^6^A regulators from public databases may enhance our understanding of the potential role of m^6^A regulators in AML and provide more effective treatment strategies.

Previous studies have indicated that AML is highly dependent on the bone marrow (BM) microenvironment that influences the survival, proliferation and therapeutic resistance of AML cells [48,49]. However, the function of m^6^A regulators on the infiltration of immune cells in the TIME of AML remained unclear. In this study, we determined three m^6^A methylation modification patterns based on 22 m^6^A regulators in AML patients. Our results revealed that many m^6^A regulators were differently expressed in the three groups. And these three patterns showed markedly distinct immune cell infiltration characteristics. M6Acluster-A was featured by adaptive immune cell infiltration and immune activation, responding to an immunoinflammatory phenotype. Patients with this m^6^A modification pattern showed a matching survival advantage. M6Acluster-B was classified as an immune-excluded phenotype, characterized by the activation of innate immunity, whereas m6Acluster-C was characterized by immune suppression, corresponding to the immune-desert phenotype. The immune-inflamed phenotype is shown by abundant immune cell infiltration in TME [50]. The immune-excluded phenotype also showed many immune cells, but they stayed in the matrix surrounding tumour cell nests and did not penetrate their parenchyma [51]. In addition, the risk score presented a high correlation with immune cell infiltration in TIME. Our findings suggested that m^6^A regulators may play an indispensable role in the formation of a complex TIME in AML patients.

In the present study, six m^6^A regulators were selected to develop a prognostic signature, and the risk score displayed satisfactory performance in predicting the survival of AML. Patients with low-risk scores had higher survival rates than those with high-risk scores. Importantly, the predictive prognostic value of the risk score was verified in another AML cohort from the GEO database. Multivariate Cox regression analysis illustrated that the risk score was an independent prognostic indicator for AML patients, consistent with the ROC curve analysis results. In addition, we also found that the risk score may act as an independent prognostic indicator of TMB for AML patients. Based on these findings, we believe that identifying risk signatures associated with m^6^A regulators can properly predict the prognosis of AML patients, thereby facilitating the selection of individual treatment strategies.

In our study, we examined the role of m^6^A regulators in the prognosis of AML patients. Differential expression analysis showed that *METTL14, YTHDC2, ZC3H13, RBM15* and *LRPPRC* were significantly differentially expressed in AML and normal samples. Patients with higher levels of *METTL14* and *YTHDC2* tended to have better survival, while patients with higher levels of *ZC3H13* and *RBM15* were related to worse outcomes in AML. Studies showed that *METTL14* acts as a tumour suppressor gene in hepatocellular carcinoma (HCC) [52] and colorectal cancer (CRC) [53]. Weng H et al. [54] reported that *METTL14* promotes AML development and the maintenance and self-renewal of leukaemia stem/initiation cells. Yang Li et al. [55] found that *YTHDC2* is correlated with immune infiltration, which may become a potential marker for head and neck squamous cell carcinoma (HNSCC) prognosis and immune infiltration. It has been reported that *METTL14* and *ZC3H13* act as tumour suppressor genes and predict poor prognosis in breast cancer [56]. *RBM15* emerges as an oncogene in leukaemia [57]. Currently, the mediation of the m^6^A regulators on patient prognosis and therapeutic response in AML remains largely unclear. Li et al [58] found that the loss of *METTL3* or *METT14* triggers T cell proliferation and differentiation disorders, thereby reducing interleukin-7 (IL-7) sensitivity in vivo. Han et al. [25] reported that silencing *YTHDF1* could boost antigen presentation, initiate antitumor responses and improve the therapeutic effect of *PD-L1* checkpoint blockade. Besides, patients can benefit from immunotherapy due to the selective removal of m^6^A regulators in TIME [59]. These results suggested that m^6^A regulators may influence the regulation of TIME to some extent. Our research also observed that *METTL14, YTHDC2, ZC3H13* and *RBM15* were closely related to immune cell infiltration. The TIDE prediction score is an effective method for immunotherapy prediction [60]. We performed TIDE algorithm to predict the relationship between these four m^6^A regulators and immunotherapeutic response, it was found that the *METTL14*-low group had a higher TIDE prediction score than the *METTL14*-high group and *ZC3H13*-high group had a higher TIDE prediction score than the *ZC3H13*-low group. All these data suggested that *METTL14* and *ZC3H13* had the potential to predict the response to immunotherapy. The discovery of these genes may provide new prognostic indicators and novel targets for treating AML.

However, our study has several limitations. First, the sample size in our study was relatively small. It is required to further improve the richness of data in future exploration. Additionally, the molecular mechanisms of m^6^A regulators in the immunotherapy and TIME of AML were not carried out.

In conclusion, our study systematically evaluated the expression, role in the TIME, and potential regulatory mechanisms of m^6^A regulators in AML. We also constructed the prognostic signature based on six m^6^A regulators to predict the survival of AML, and the risk score was identified as an independent prognostic indicator for AML. Importantly, we screened out four prognosis-related m^6^A regulators, assessed their value in the immunotherapy of AML and verified the expression levels of these four m^6^A regulators in AML patients with different risk categories. In brief, our results provide novel insights into the potential role of m^6^A regulators for prognostic prediction and therapeutic targets for AML patients.

## Conclusions

Our study explored the distinct molecular landscape in different m^6^A modification patterns, including pathways, relationship with immune cell infiltration and prognostic value. We also screened for risk signalling of m^6^A regulators in AML and constructed and verified the predictive dependability and sensitivity of the risk prediction model. The results provided new insights into the potential roles and mechanisms of m^6^A regulators in AML. Through the study, identifying m^6^A regulators related to prognostic prediction and immune responses may provide novel targets for improving immunotherapy and prognosis for AML patients.

## Supplementary Material

Supplemental Material

## Data Availability

All data used in the study will be shared on reasonable request to the corresponding author. The datasets analysed during the current study were derived from the following public domain resources: TCGA https://portal.gdc.cancer.gov/ ; GSE9476 https://www.ncbi.nlm.nih.gov/gds/?term=GSE9476; GSE23312 https://www.ncbi.nlm.nih.gov/gds/?term=GSE23312; UCSC https://xenabrowser.net/datapages/

## Highlights

- Comprehensive analysis of genetic variation landscape of m^6^A regulators in AML.
- Three distinct m^6^A modification patterns were determined, which have different pathway enrichments, characteristics of immune cell infiltration and prognostic values.
- The risk score based on the m^6^A regulators-associated prognostic signature is an independent prognostic factor in AML
- We screened out four m^6^A regulators (*YTHDC2, METTL14, RBM15* and *ZC3H13*), which were associated with response to immunotherapy in AML.
