# Supplementary material for: m6A RNA methylation regulators predict prognosis and indicate characteristics of tumour microenvironment infiltration in acute myeloid leukaemia
Source: Epigenetics. 2022 Dec 25;18(1):2160134. doi: 10.1080/15592294.2022.2160134 (PMC9980463; doi:10.1080/15592294.2022.2160134)
Supplement: Supplemental Material [file KEPI_A_2160134_SM9602.zip › supplement/Supplementary Information.docx]

**Supplementary Information**

**Figure S1. Identification of consensus clusters of the 22 m^6^A regulators.** Delta area curves (A)and tracking plot (B) with k =2 to 9. (C)Principal component analysis of three determined m^6^A clusters. (D) Heat map and clinicopathologic features of the three m^6^A modification patterns

**Figure S2. Biological characteristics of each modification pattern.** (A–C) GSVA of distinct m^6^A modification patterns reveals the activation states of biological pathways in each pattern. The heat map shows biological processes, with red representing activated pathways and blue representing inhibited pathways. (A) m6Acluster-A versus m6Acluster-B; (B) m6Acluster-B versus m6Acluster-C; (C) m6Acluster-A versus m6Acluster-C. (D) Abundance of immune-infiltrating cells in TME in the three m^6^A modification patterns. The lower and upper ends of the boxes represent the interquartile range of values. The lines in the boxes represent the median value, whereas the dots represent outliers. The asterisks denote the statistical *p*-value (**p* < 0.05; ***p* < 0.01; ****p* < 0.001)

**Figure S3. Verification the prognostic value of m^6^A regulators in the GEO database.** (A-D) Survival analysis was performed for subgroups of patients stratified by *YTHDC2* (A), *METTL14* (B), *RBM15* (C) and *ZC3H13* (D) expression (*p<* 0.05, log-rank test)

**Table S1. Details on three risk category groups of AML patient samples**

**Table S2. Primers for quantitative RT-PCR**

**Table S3. Six m^6^A regulators were identified as the independent prognostic genes**
