# Supplementary material for: m6A RNA methylation regulators predict prognosis and indicate characteristics of tumour microenvironment infiltration in acute myeloid leukaemia
Source: Epigenetics. 2022 Dec 25;18(1):2160134. doi: 10.1080/15592294.2022.2160134 (PMC9980463; doi:10.1080/15592294.2022.2160134)
Supplement: Supplemental Material [file KEPI_A_2160134_SM9602.zip › supplement/Supplementary Table 1 1st revision.docx]

Details on AML patient samples

| Risk category | NO. | Gender | Age | WBC(×10^9^/L) | FAB | Genetic abnormality |
| --- | --- | --- | --- | --- | --- | --- |
| Fevorable | #16 | Male | 46 | 10.97 | M2 | 46, XY t (8;21) (q22; q22)  [5]/46, xy[3] |
|  | #17 | Female | 48 | 36.54 | M5 | Mutated CEBPA |
|  | #18 | Male | 42 | 69.98 | M5 | CBFb-MYH11 |
|  | #19 | Male | 48 | 51.54 | M5 | 46, XY t (8;21) (q22; q22) [4]/46, xy[2] |
|  | #20 | Female | 40 | 10.73 | M5 | Mutated CEBPA |
| Intermediate | #21 | Female | 49 | 1.67 | M5 | Mutated IDH1; NARS |
|  | #22 | Male | 14 | 5.9 | M5 | Mutated NARS |
|  | #23 | Male | 39 | 108 | M5 | Mutated KIT |
|  | #24 | Female | 29 | 1.16 | M1 | Mutated IDH1; VAV1-MYO1F |
|  | #25 | Female | 62 | 61.35 | M5 | MLL-AF9 |
| Adverse | #26 | Male | 48 | 198.99 | M5 | Mutated FLT3- ITD;  CEBPA; NRAS; TP53; WT1 |
|  | #27 | Male | 14 | 12.71 | M5 | Complex karyotype |
|  | #28 | Male | 45 | 153.8 | M1 | Mutated ASXL1 |
|  | #29 | Male | 49 | 8.62 | M5 | Mutated TP53 |
|  | #30 | Female | 65 | 21.68 | M5 | Mutated TP53; BCOR |
