# Supplementary material for: m6A RNA methylation regulators predict prognosis and indicate characteristics of tumour microenvironment infiltration in acute myeloid leukaemia
Source: Epigenetics. 2022 Dec 25;18(1):2160134. doi: 10.1080/15592294.2022.2160134 (PMC9980463; doi:10.1080/15592294.2022.2160134)
Supplement: Supplemental Material [file KEPI_A_2160134_SM9602.zip › supplement/Supplementary Table 2 1st revision.docx]

**Primers for quantitative RT-PCR**

| METTL14-F(homo) | TGGACCTTGGAAGAGTGTGTT |
| --- | --- |
| METTL14-R(homo) | GTGCTACGCTTCACAGTTCC |
| ZC3H13-F(homo) | AGATGACGAGTCCAAGTTAGATGA |
| ZC3H13-R(homo) | GGCATAAGACCAGACCAATCC |
| RBM15-F(homo) | GCAGTCCAGAATTGAGCAGTAG |
| RBM15-R(homo) | TACCTCGTCTGTCTCTGATTGG |
| YTHDC2-F(homo) | AATCTCCATCTCCAGCATTACAC |
| YTHDC2-R(homo) | AGAAGGACTAGCACAAGGACTT |
