# Supplementary figures and images for: m6A RNA methylation regulators predict prognosis and indicate characteristics of tumour microenvironment infiltration in acute myeloid leukaemia
Source: Epigenetics. 2022 Dec 25;18(1):2160134. doi: 10.1080/15592294.2022.2160134 (PMC9980463; doi:10.1080/15592294.2022.2160134)

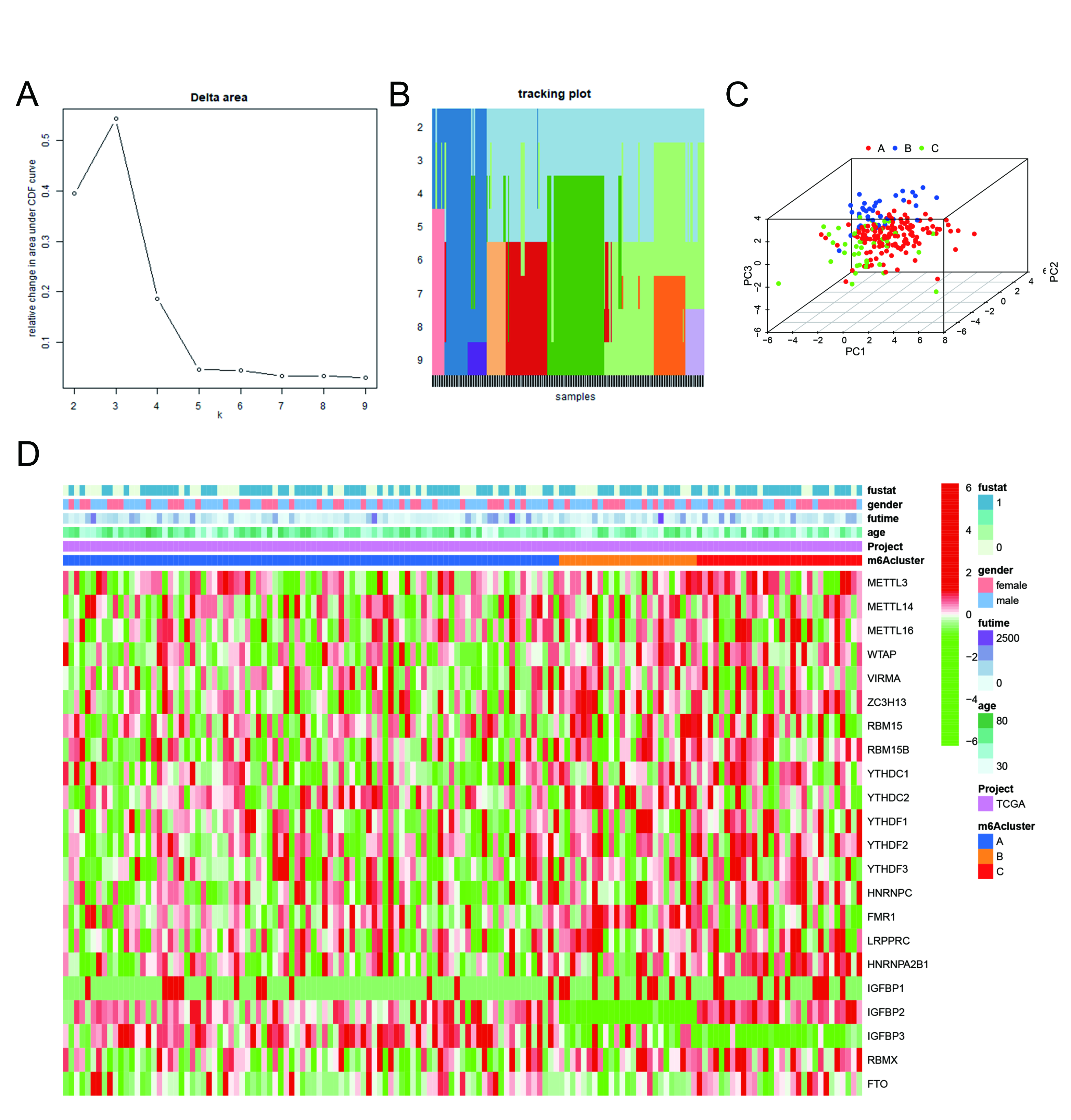

Supplement: Supplemental Material [file KEPI_A_2160134_SM9602.zip › supplement/Supplementary Figure 1 1st revision.tif]

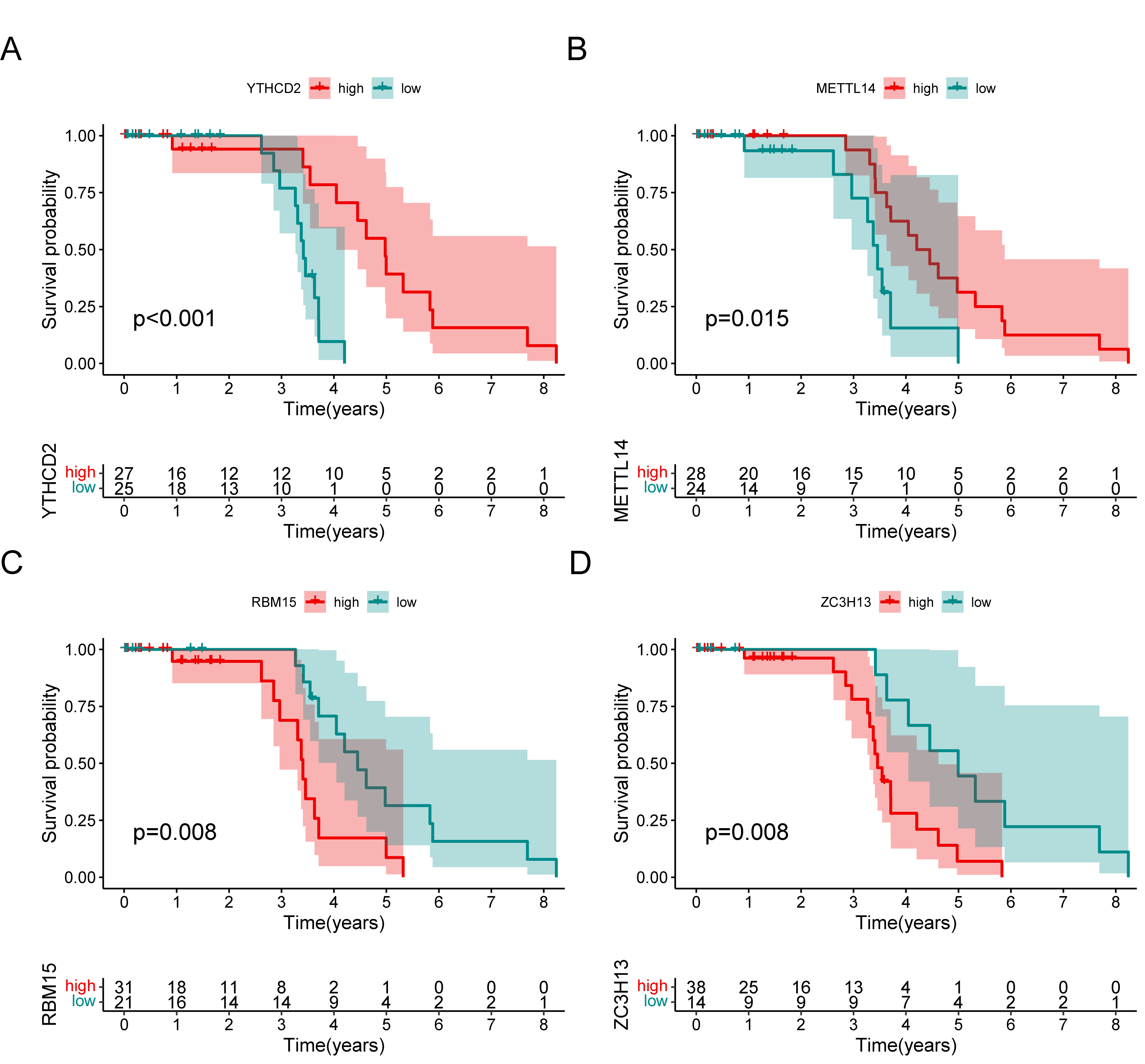

Supplement: Supplemental Material [file KEPI_A_2160134_SM9602.zip › supplement/Supplementary Figure 3 1st revision.tif]
